# On the Potential of Packaging for Reducing Fruit and Vegetable Losses in Sub-Saharan Africa

**DOI:** 10.3390/foods11070952

**Published:** 2022-03-25

**Authors:** Lionel D. S. Tapsoba, Sountongnoma M. A. Kiemde, Bernard F. Lamond, Julien Lépine

**Affiliations:** 1 Faculty of Business Administration, Department of Operations and Decision Systems (ODS), Université Laval, Québec, QC G1V 0A6, Canada; kiemde.anicet97@gmail.com (S.M.A.K.); bernard.lamond@fsa.ulaval.ca (B.F.L.); julien.lepine@fsa.ulaval.ca (J.L.); 2 Centre Interuniversitaire de Recherche sur les Reseaux d’Entreprise, la Logistique et le Transport (CIRRELT), Montreal, QC H3T 1J4, Canada

**Keywords:** food waste, food packaging, bio-sourced packaging, fruits and vegetables, Sub-Saharan Africa

## Abstract

Access to food remains a critical issue in Sub-Saharan Africa. In fact, 24.1% of its population suffers from undernourishment, and malnutrition affects more than a third of children under five years old. This problem will be exacerbated as the Sub-Saharan African population is predicted to double by 2050. To address this problem, it is imperative to meaningfully improve accessibility of fruits and vegetables for the population.They are an excellent source of vitamins and minerals that can fight malnutrition. Fruit and vegetable accessibility can be improved by reducing losses, which are estimated on average to be 50%. A literature review shows that there are many areas where solutions can be implemented to reduce these losses. These areas, in order of decreasing occurrence in the literature are: Cold storage, harvesting methods and pre-storage treatments, packaging, transport to markets and the sale stage. The reduction of food waste in SSA involves the establishment of better practices in all these areas. After analysis, it emerges that packaging should generate more interest due to its comparative ease of implementation to support other technologies like cold storage. Packaging made from agricultural waste or non-consumable materials should be highlighted to prevent pollution issues. This solution, in addition to offering a strong potential to fight against pollution, could also increase farmers’ income.

## 1. Introduction

About 21.4% of the population of Sub-Saharan Africa (SSA) suffered from undernourishment in 2020 [1]. This problem is more critical since, in fact, the population does not only suffer from insufficient access in terms of quantity of food but also, and most notably, in terms of quality of food. The latter is called “hidden hunger”. Indeed, a study in 32 countries in SSA from 2006 to 2016 about malnutrition of children under five years old indicated the magnitude of the problem [2]. According to this study, around 33.2% of children were suffering from growth retardation, 7.1% from emaciation (low weight-to-age ratio) and 16.3% from underweight.

Moreover, malnutrition was directly or indirectly responsible for the death of 54% of children under five years old in developing countries in 2001 [3]. The principal cause of this situation is the lack of nutrients and minerals in diets. In fact, the food staples of Africans are only based on one or more of the three basic food groups: Grains, roots and tubers. Combining these foods with fruits and vegetables can reduce the prevalence of malnutrition [4].

In order to achieve the United Nations Sustainable-Development Zero-Hunger Goal (SDG2) and the Good-Health and Wellness Goal (SDG3), increasing fruit and vegetable production is necessary to meet global nutritional needs [5].

Current trends indicate that this situation will be exacerbated in the future. Indeed, according to United Nations projections, the population in SSA will increase by 108% by 2050 [6]. In addition, the percentage of people suffering from hunger in SSA in 2030 will increase by between 6 and 8%, depending on the region. Reducing food insecurity in SSA is a crucial issue. As stated before, we must focus not only on the quantity but also on the quality of the nourishment offered. Increasing fruit and vegetable access can therefore ensure a rich and varied diet for the populations and can reduce the rate of malnutrition.

Despite the importance of fruits and vegetables to a healthy diet, most of the food studies and the literature reviews on food in SSA have focused on other crops [7]. Increasing interest and the number of interventions for fruit and vegetable access are therefore essential to reduce the prevalence of malnutrition in SSA. While Africa has the most unexploited arable lands, simply increasing the area of cultivated land will not be enough to solve the problem. The demographic expansion will be accompanied by an increase in land demand, reducing the availability for agriculture. Increasing production to fulfil demand will also have a significant impact on the environment through greenhouse emission and deforestation [8,9]. Hence, one of the first steps to mitigate these problems also involves reducing food losses in order to slow down the increasing demand for new production.

Most fruit and vegetable losses in SSA occur before consumption. These losses are estimated to be 55.9 ± 25.4% and 43.5 ± 16.6% for fruits and vegetables, respectively [10]. Additionally, eliminating these losses could increase the total value of vegetable supply by 45% (US $72 million) per annum and also reduce vegetable import needs by 22%, which amount to 127,000 tons [11].

Various approaches and solutions have been proposed and tested, but some of them are based on inappropriate technologies and do not have the expected impact in real-life applications. From all the solutions analyzed in a large study conducted in Southeast Asian and SSA countries [12,13], solutions focusing on food packaging seem to have the greatest potential.

This paper will investigate in detail whether packaging is the most appropriate solution. The most common solutions in the literature on the reduction of losses will be presented in this article, and their feasibility will be studied. The efficiency of packaging as a solution will also be studied. Next, recommendations for optimal packaging efficiency as well as loss reduction will be presented.

## 2. Literature Review

It is difficult to estimate precisely the loss quantity for fruits and vegetables in SSA due to the limited data availability and the limited number of resources in the literature [10]. Fortunately, some research has been conducted to determine the magnitude of losses and propose solutions. Gustavson et al. [14] describe the issue well; the magnitude of fruit and vegetable losses in SSA in percentage of the total mass is 10% before harvest, 8% during harvest, 20% during processing, 10% during distribution and 2% at consumption stage, for a total loss of about 50%. Different statistics relating to fruit and vegetable losses in SSA, that were obtained from different authors, are presented in Table 1. For Sheahan and Barrett [15], the main causes of losses in SSA are poor handling of fruits and vegetables and inadequate storage and transport processes. They also pinpoint large on-farm losses and they raise issues about economic decision-making such as maximizing profit versus minimizing losses.They recommended the installation of cooling systems for the storage and transport of food and for avoiding overloading of vehicles. The use of a cooling system can slow down the rotting processes of fruits and vegetables as well as the proliferation of sprouts. Overloading vehicles increases the mechanical pressure on fruits and vegetables, which leads to their physical degradation. For the losses incurred on the farm, which amount to 20%, these can be reduced by up to 4% when the various measures recommended in the literature are correctly applied. They also mention that on-farm technologies are well referenced in the literature and that attention should also be paid to off-farm interventions to achieve the goal of reducing losses [15].

The results of a study conducted in Ghana reveal loss statistics for cabbages contained in large bags [12]. These bags were damaged and they could not offer adequate protection. The losses due to physical damage were on average 32% of received mass with a standard deviation of 26% during the wholesale, and an average of 45% of received mass with a standard deviation of 28% during the retail sale. In Rwanda, losses for bananas were on average 15% with a standard deviation of 21% at the farm. These bananas were transported without packaging (the bunches were placed directly in the trucks) and sorted before the sales stage. The losses recorded were on average 35% of received mass, with a standard deviation of 33% for wholesale sales, and an average of 30% received mass with a standard deviation of 24% for retail [12].

A study on tomato production and sale in SSA suggests that most of the losses are due to poor road conditions during transport, which generates a lot of physical damage on the freight [21]. The lack of suitable storage infrastructure such as cold rooms also worsens tomato losses. According to the authors, improving the strength of the packaging and reducing the weight of food in transport boxes can help reduce the physical damage to tomatoes during transport. According to a study in Uganda, 15% of all plantain produce experiences some level of deterioration along the supply chain (7% of bananas deteriorate completely and have no residual value, while 8% partially deteriorate and are sold at reduced prices) [20]. The use of boxes would provide protection against impacts and thus could minimize physical and economic losses.

In Ethiopia, the physical losses for plantains along the supply chain were evaluated at 26%. Most of these losses occur during retail due to loss in quantity and quality of plantains, with losses during sales being 16% [20]. Another study estimated Ethiopia’s post-harvest banana losses in the supply chain to be 26%, most of which is at the retail market level (2/3) and wholesale trade (1/3); mechanical damage was the main cause for banana loss at the wholesale level [16].

A study on losses in Ethiopia was conducted about papaya.The papaya post-harvest losses were 22%, 12% and 9% during wholesale, transportation and storage, respectively. The main cause of fruit losses was fruit softening, rotting, wounding and compacting due to inappropriate transporting, storage conditions and lack of appropriate marketing place [18].

Tomatoes losses at the wholesale stage in Nigeria were estimated at 23.3% and the amount of mechanical damage (physical deterioration of fruits due to shocks and vibrations) in the evaluated sample was 23% according to a study by [19].

According to another study, the losses of tomatoes in Nigeria at the retail level were 20% with a standard deviation of 10%. Post-harvest losses are between 30 and 50% of the initial mass [17].

## 3. Methods

The FAO defined the notions of “food loss” and “food waste” in their methodology as follows [14]: “Food losses refer to a decrease in food quantity or quality in the early stages of the food supply chain, reducing the amount of food suitable for human consumption. The concept food losses are thereby often related to post-harvest activities with lacking system or infrastructural capacities. Food waste on the other hand often refers to later stages of the food supply chain, such as retail and consumer households. Hence, the causes of food waste are often related to human behavior.”

As noted by the same authors, the methodology of the studies “is challenged by major data gaps for both waste percentages of losses and waste and the causes of losses and waste. The results must therefore be taken with great caution”. Furthermore, this definition has its own limitations because some losses in the early stages of the supply chain such as harvesting can be voluntary (destruction of certain fruits due to their physical aspects or for economic reasons). To avoid this ambiguity, the term food losses will be used to refer to both food losses and waste.

In order to determine the most proposed stage of interventions for reducing fruit and vegetable losses in SSA, a meta analysis based on 42 articles was conducted. From these, 37 articles are part of the Postharvest loss reduction intervention database (2020). This database is from a study carried out in 57 countries in SSA and South Asia from 1970 to 2019 in which 12,907 studies were filtered to form a base of 334 suitable studies [7].

We used this database of 334 articles to select 37 articles according to two main criteria: The country and the crops.

As to the countries, we retained the articles that concern Benin, Burkina Faso, Ghana, Cameroon, Togo, Ivory Coast, Democratic Republic of the Congo, Ethiopia, Guinea, Kenya, Gambia, Niger, Malawi, Mali, Mauritania, Mozambique, Nigeria, Sierra Leone, Somalia, South Africa, Sudan, Tanzania, Uganda, Zambia, Zimbabwe. For crops, we have selected articles concerning fruits and vegetables, namely banana, mango, leafy vegetables, tomatoes, citrus fruits, onion, potato, papaya, sweet potato.

We updated this database by incorporating recently published articles on fruits and vegetables in SSA, bringing the total number of articles to 42. The five articles that we added were identified on the basis of keywords. The resulting database covers a period from 1970 to early 2022. Only articles published before February 2022 whose studies targeted one or more well-identified African countries were selected.

**Figure 1:**
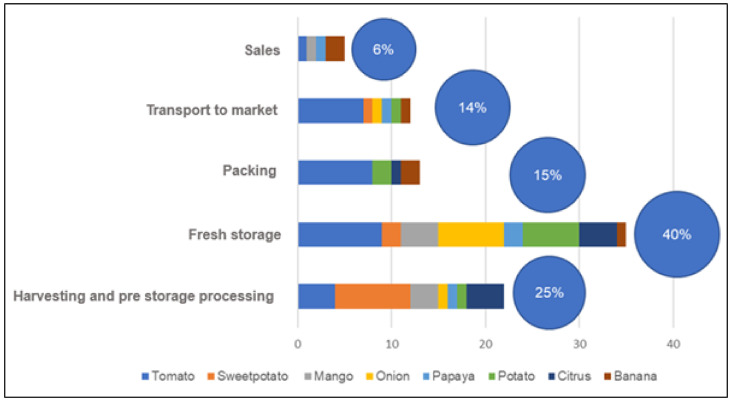
Number of studies on fruit and vegetable loss reduction in Sub-Saharan Africa by areas of intervention.

Figure 1 shows the results of this analysis where the different intervention areas are:

- Harvesting and pre-storage processing: This category includes the picking of fruits and vegetables and all the steps before storage (e.g., harvesting for any stage of maturity, handling, curing and further drying).
- Fresh storage: This category includes storage of processed food at the farm, during transport and at markets. In some cases, there is no cold storage in the chain.
- Packing: Process of sorting, grading and putting fruits and vegetables in their packaging before transport.
- Transport to market: Transport of fruits and vegetables to the various markets (retailers and wholesalers).
- Sale: Phase of sale of fruits and vegetables (wholesale and retail) after transport from farms to markets and inter-market transport.

## 4. Results and Analysis

The database analysed showed that the most mentioned intervention areas in the articles are about fresh storage (40%), harvesting (25%), packing 15%, transport (14%) and sales (6%).

Another thing that the analysis of the different databases and literature reviews on the food losses revealed is the lack of data on the status of fruits and vegetables in SSA compared to cereals. Indeed, of the 334 studies on SSA losses presented in the Postharvest loss reduction interventions database, only 7.5% of the interventions focused on fruits and 6.2% on vegetables (non-cumulative) compared to 54.9% on cereals [7]. These results are similar to those presented in the SSA loss reviewed last year in which only 10% of the 213 articles identified addressed fruits and vegetables [10]. These results show that fruits and vegetables are not given enough attention in SSA compared to cereals. This interest can be explained by the higher calorific value of cereals. Emphasizing cereals can be an effective strategy to fight hunger by helping to meet the body’s energy needs. However, fruits and vegetables are essential and their under-consumption leads to malnutrition problems in SSA [2,5,18].

### Critique and Analysis of Proposed Solutions

**Figure 2:**
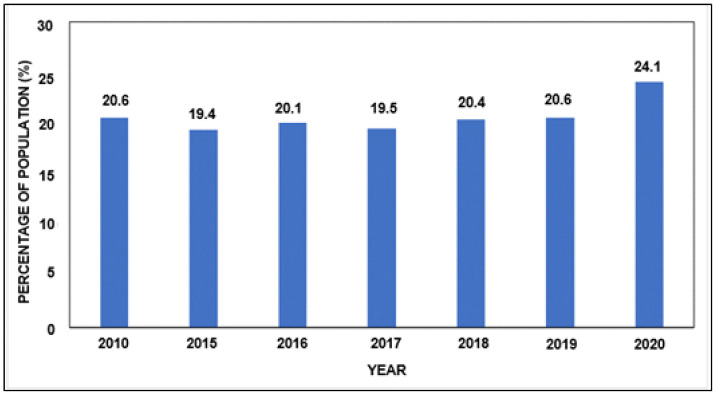
Prevalence of undernourishment in SSA between 2010 and 2020 (Source: FAO database).

According to the data collected by the FAO [1], there has been no significant reduction in the proportion of hungry people in SSA over the last decade (Figure 2). Furthermore, the latest projections suggest an increase in the proportion of hungry people in SSA due to COVID-19 [1]. Other causes of this increase are wars and insecurity as well as refugee relocations that have increased during this decade [22,23]. SSA is also a region that is particularly sensitive to climate change and is prone to droughts, which makes it more difficult for people to access food (increasing soil pressure, difficulty accessing water …) [24,25].

However, while there are several causes of the hunger situation in SSA, the solutions most commonly found in the literature related to food waste may not be the most appropriate. Indeed, their implementation in SSA is fraught with difficulties. It is important to rethink these solutions in order to make their application more effective and efficient in SSA when considering the small number of articles related to fruit and vegetable loss reduction in SSA discussed previously.

The area of intervention that stands out the most from the meta analysis conducted on fruit and vegetable losses in SSA is fresh storage (40% of the papers reviewed).

Cold storage is the primary method to preserve fruits and vegetables. In fact, without proper cooling, losses can hardly be reduced. While their effectiveness has been demonstrated in other parts of the world, investing in cold-storage solutions that require electricity is therefore inappropriate for SSA countries [26]. However, there are more and more studies related to fresh-storage solutions that do not require electricity and are therefore more adapted to SSA countries and for small farm holders [27]. These cold storage solutions are for instance hydro cooling, evaporative cooling and forced air cooling. While these solutions are not as effective as the electricity-based ones, they show interesting results for reducing fruit and vegetable losses in developing countries [13]. Cold storage is a method of slowing down the decomposition of fruits and vegetables, but it does not prevent the causes of fruit spoilage, especially shocks and vibrations occurring at different stages but mostly during transport. In addition, most farm holders in SSA have low incomes and this solution is particularly expensive in terms of initial investment and operating costs. In addition, only 43% of the population in SSA has access to electricity and more than 80% of people in rural areas live without electricity [26].

As far as we can see, this situation will remain a challenge for years to come as SSA has the lowest electrification growth rate in the world. The average growth is 0.8% per year compared to 2.4% per year for other developing countries [26].

Cold storage can extend the conservation of fruits and vegetables during storage but does not prevent against the causes of spoilage such as mechanical damage due to shocks and vibrations. Packaging should be combined with cold storage in order to reduce fruit and vegetable losses in SSA since there are two different aspects of postharvest causes.

Another alternative would be to reduce the travel time between farms and markets by improving and building more roads. Indeed, according to several studies, losses during transport to markets are due to mechanical damage to the fruit from the condition of the roads [13,15,28]. SSA is known to be the region with the least developed transport infrastructure. The roads are generally in poor quality and conditions and road-transport costs are very high compared to other regions [29]. While funding for road infrastructure has increased significantly in recent years, the development of road networks remains prohibitively expensive. Moreover, investments go mainly into road construction, connecting major centres while mainly rural agricultural areas do not benefit from these investments [29].

**Figure 3:**
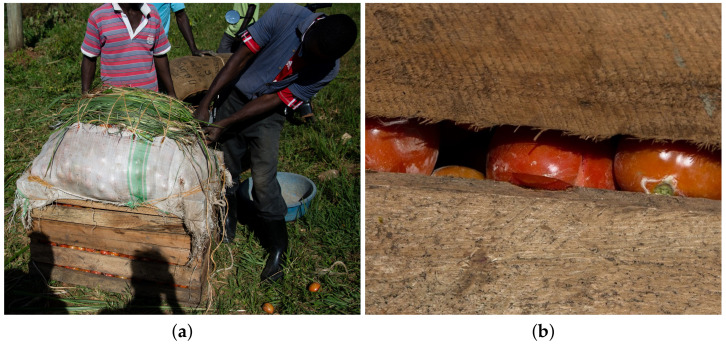
Tomato packaging in Uganda farm (©Julien Lépine). (**a**) Tomato packaging for transportation. (**b**) Zoom in on mechanical damage that occurred during packing.

**Figure 4:**
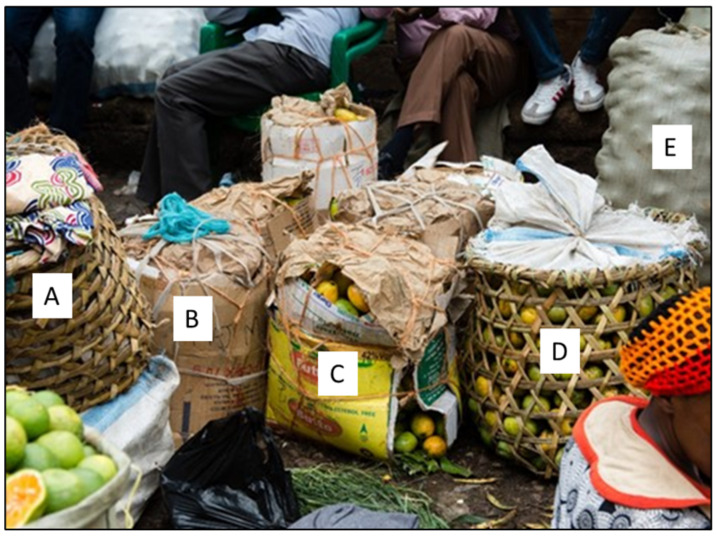
Different types of packaging used in a Uganda market: A and D show raffia basket, B and C cardboard and E plastic bag. (©Julien Lépine).

The development of adequate transport infrastructures is essential for optimal transport of fruits and vegetables. The use of adequate packaging can help reduce the consequences of the lack of road-network developments with an affordable cost, even if packaging cannot replace the construction of roads. Figure 3a shows the current packaging used in a small farm in Uganda for tomato transport by motorcycle. The farm holder used a wooden box with a plastic bag on the top to increase the capacity, which overloads the box. Leafs were added to reduce the pressure of the rope on the tomatoes. On Figure 3b, the zooming shows the mechanical damages that occurred during packaging due to the overload and the roughness of wooden box. Figure 4 shows different packaging used in a Uganda market. Figure 4A,D show raffia baskets, Figure 4B,C cardboard and Figure 4E plastic bags. Most of these packagings are inadequate and do not offer protection. This figure reveals the lack of financial resources and the necessity to offer affordable and adequate packaging for farm holders and sellers.

Among the areas of intervention found in the literature such as cold room, adequate harvesting methods, refrigerated vehicle to preserve the cold chain or the implementation of appropriate policy for the agricultural sector, packaging may be the solution with the most potential for reducing fruit and vegetable losses in terms of simplicity of implementation, initial investment and cost efficiency.

## 5. Discussion—Packaging for Reducing Fruits and Vegetables Losses in SSA

### 5.1. Effectiveness of Packaging as a Solution for Reducing Fruit and Vegetable Losses in SSA

In this paper, the term packaging designates any material used to contain a product for the purpose of protection, transport and storage. For example, boxed cases carrying bunches of bananas, plastic bags with onions or a basket with oranges are different types of packaging. Packagings can be classified according to their constitutive materials. A distinction is made between plastic, cellulosic (e.g., lignite, pulp and cellophane), ceramic and metal packaging [30,31]. Packaging is said to be biodegradable if it degrades in the environment under certain conditions. It is said to be bio-based or bio-sourced if it comes from natural products (e.g., agricultural byproducts, plants or wood). The right choice of packaging is based on certain performances which can be expressed in chemical and physical terms. It is also important to consider the interaction between the packaging materials and the food transported. The aesthetic appearance of the packaging can also be a selection criterion. Finally, the choice of packaging can also be made considering its ecological impact. Since the main goal is the reduction of losses, good packaging can be defined as packaging with good chemical and physical performance that allows it to preserve food well while being environmentally friendly.

There are only a few studies and experiments that can be used to evaluate the potential of packaging for fruit and vegetable loss reduction in SSA. A study conducted in India shows that the use of plastic crates resulted in a 12% decrease in breakage during transport of guava compared to the use of unlined plastic crates [13]. The presence of the liner reduced vibration damage and absorbed shocks during transport. Similar results were observed in Rwanda and Ghana where wood boxes or woven liner baskets were used [13]. Furthermore, a comparative study between raffia baskets (the most commonly used basket) and plastic baskets conducted on tomatoes transported by trucks in Nigeria shows that plastic baskets reduced transport damage by 5% compared to raffia basket. On average, 41% of the tomatoes were damaged when transported in a raffia basket [28].

As mentioned in a study, the use of adequate packaging during storage and transport of fresh fruits and vegetables has a positive impact on the quality and shelf life of these products. Between 30% and 50% of fruits and vegetables are lost when transported in traditional packaging in SSA and these losses can be reduced by using more effective packaging [32].

In addition to protecting the fruit and vegetables, packaging could also serve a marketing function that increases vendor revenue and allows for product traceability. Moreover, the use of agricultural waste for the design of bio-sourced packaging using agricultural byproducts such as banana or papaya leaves can also reduce losses and generate income through waste valorization. Another advantage of the use of more effective packaging is they could increase the shelf life of fruits and vegetables in the markets. In fact, by preserving the physical integrity of fruits and vegetable, packaging could contribute to preserving the natural physical barrier against external agents. This makes these commodities more available to the population.

Packaging can have other functions that preserve food, such as limiting water nutriment losses. A study analyzed the effect of various plastic film packagings on tomato nutritional values [33] . The results showed that after eight days at 20 °C, the packaged tomatoes retained most of their nutritional qualities compared to unpackaged ones. Differences were also observable between the results of different plastic films. Some wraps that prevented evaporation resulted in the fruit not reducing in mass [33].

Another study on the use of Modified Atmosphere Packaging (MAP) showed that it improved shelf life and preserved the quality of African nightshade. The use of MAP reduced weight loss from 76% to 84% at 20 ${}^{\circ }$ C compared to controls. In addition, it maintained protein, vitamin C and E, β-carotene, lutein, lycopene and chlorophyll content compared to control samples [34].

These results show that packaging allows reduction in waste. It also preserves the nutritional values of fruits and vegetables, which are essential in the fight against invisible hunger. However, the implementation of some nutrient-preserving packaging technologies in SSA requires investment and could not be sustainable due to high cost and unavailable specific-material supply.

### 5.2. Analysis and Discussion on Packaging Solutions

Our findings show that there is a lack of studies concerning loss reduction for fruits and vegetables in SSA. Moreover, to reduce food waste in SSA, appropriate and low-cost solutions to increase the accessibility of fruits and vegetables for the populations of this region of the world should be investigated.

Because of their simplicity and low cost, packaging solutions have a great potential to fight against food loss and waste in SSA. Indeed, they allow facing the challenges related to the lack of good roads and energy infrastructure in SSA, which hinders the implementation of other solutions. While the packaging can be implemented during on-farm storage and the same packaging can be used during transportation and sale stage to reduce losses in different stages.

Field trials about postharvest technologies have been conducted by Saran et al. [13] in SSA and South Asian countries. These trials focused on the effectiveness in terms of cost-benefit ratio for 21 cases of improved handling, packing, storage and processing. This preliminary trial showed that most technologies are appropriate and improved packagings have very short payback period (immediately to three months). For cold storage, the zero-energy cool chamber (ZECC) or CoolBot technology may require between five months and more than one year, depending on accessibility or availability of infrastructure and equipment. All theses solutions are cost effective; however, considering the initial cost and the pay back period, packaging could be a solution generating more interest to help reduce losses. Even if its impact is less than cold storage, given the urgency of the situation, its speed of implementation can make it an asset to help against food loss and support small farmers before the technologies of cold storage are profitable [13].

However, implementation of packaging solutions in SSA can be limited by their cost, even though it is less expensive than other solutions such as building roads and it could be used to mitigate the lack of infrastructure. Moreover, local food-supply chains in SSA are made such that the packaging cannot be easily returned to the farm holders or carriers making investment on such solutions more complicated. In fact, in the world, 79% of plastic is dumped into the landfills or on the environment, 12% is incinerated and 9% is recycled [35].

Improving bio-based packaging such as those made from agricultural waste and byproducts that are traditionally used in these regions (Figure 4) could be a solution to these problems since this type of raw material is cheaper than synthetic materials. Moreover, by employing the material traditionally used, the packaging would benefit the local expertise and local supply chain. Developing packaging solutions made from local material (traditional or not) will develop and improve local expertise. It will also benefit the local economy by promoting traditional packaging.

Moreover, the use of agricultural waste for packaging would allow for waste valorization [36]. The development of sustainable packaging like bio-based packages, in addition to reducing pollution, would be an additional source of direct revenue (sale of packaging) and indirect revenue (loss reduction and produce sale increase) [13]. Bio-sourced packaging can also reduce greenhouse gas (GHG) emissions as they come from materials (agricultural waste) that would have been burned or buried if not used. The additional protection they provide compared to packaging currently used in SSA (Figure 4) also decreases food waste and the GHG associated with their life cycle (from seeding to decomposition).

The production of environmentally friendly packaging made from locally sourced materials can be simpler and less expensive for farmers than from plastic and other synthetic packaging. Indeed, the supply of synthetic materials can be difficult in SSA countries due to their low industrialization and high transport costs when imported [29]. As pointed out by [37], the use of eco-friendly packaging plays a very important role in preserving the environment especially in developing countries. It is better to avoid packaging made of plastic or other synthetic materials because they pose a danger to the environment and human health [38,39,40]. Using more eco-friendly packaging would reduce the hazards to individuals and the local ecosystem [36,37]. Moreover, the promotion of ecological packaging can be a vector of awareness for consumers. This awareness is essential for the fight against plastic pollution because consumers play a leading role in developing countries in the prevention of plastic pollution [38]. ome packagings based on agricultural waste have been developed such as, for example, carton boxes made by The Greenery (15% tomato plant residues and 85% recycled paper and board) a company from the USA and operated in USA, Paper Foam^®^ made by PaperFoam a Dutch company that produces the Netherlands,United States and Malaysia. This package was made by 70% of potato or tapioca residues and 15% wood-fibres [41] NPulp^®^ produced by YFY Jupiter in China (85% recycled paper and 15% wheat- and rice straw). In China, farmers burn ninety percent of the straw to clear their fields, which leads to soil degradation and air pollution. By using straw for packaging, the costs associated with combustion and pollution can be greatly reduced. These packagings were produced in the context of heavily industrialized countries such as the USA, Germany or China [41]. Smaller scale bio packagings were also developed for newly industrialized countries. For instance, the Canadian company CFK packaging developed for the Malaysian market a product called Earthcycle^®^ made from with palm fibre pulp, waste from fruit and oil production [41]. Packagings made from agricultural waste targeting even smaller markets were also studied to respond to the needs of smallholder farmers in SSA such as Kenya [42].

Thus, the adoption of bio-sourced packaging would seem to be a win–win alternative for farmers, consumers, and the planet. Overall, effective reduction of food access problems in SSA requires the implementation of simple, low-cost solutions. Adopting bio-sourced packaging with better physical properties is one such solution. Other simple, inexpensive and easier to implement solutions such as disinfecting fruits and vegetables on the farm by washing them would eliminate certain germs and preserve the fruits longer. The sanitizing of the packaging used for storage and transport as well as the respect of the maximum capacity of the boxes would make it possible to reduce the losses during transport. These types of solutions, therefore, show great potential to reduce losses and waste without requiring significant investments.

## 6. Conclusions

In SSA, 24% of the population is undernourished, and an efficient way to solve a part of this issue is to reduce food waste, as around 50% of food is lost before it reaches SSA consumers.

The main area of interventions mentioned in the literature is related to cold-storage technologies. While this solution has already been shown to be the most effective solution to reduce fruit and vegetable losses, it is not so efficient for the majority of SSA countries for two main reasons. First, to be highly effective, these solutions need a power source, but SSA has the lowest electrification in the world. Next, cold storage reduces the decay rate of produce but it does not prevent it from happening. On the other hand, protective packaging can be associated with others technologies to prevent damage from occurring during transportation and avoid the subsequent degradation. The use of packaging solutions has real advantages in terms of efficiency, initial investment cost and profitability. In addition, the use of agricultural waste for the design of these packaging solutions would valorize agricultural waste. This valorization in addition to reducing losses would be an additional source of income for farmers and local communities. They also present advantages in terms of preserving nature and the health of consumers.

This study adds to the literature on loss reduction for fruits and vegetables in SSA. It shows the importance of the problem of malnutrition in SSA and the need to provide appropriate and low-cost solutions to increase the accessibility of fruits and vegetables for the populations of this region of the world. It also underlines the interest of developing ecological packaging to fight against pollution.

## Figures and Tables

**Table 1:** Statistics about fruits and vegetables in SSA including magnitude and stages of fruit and vegetable losses present in the literature from 2012 to 2021. The table only presented statistics for papers showing results of food losses in a specific stage, specific fruits or vegetables and specific countries in SSA.

Authors	Countries	Year of Publication	Produce	Loss Stage	Magnitude	Data Source (n= Sample Size)
Abewoy et al. [16]	Ethiopia	2021	banana	Post harvest (all) Wholesale Retail	26.5% 35.9% 64.10%	Survey (wholesale n=10, retail n=15)
Bwade et al. [17]	Nigeria	2020	tomato	post harvest (all)	[30%, 50%]	Literature review
Tolessa Lemma et al. [18]	Ethiopia	2020	papaya	Wholesale Retail	21.5% 15.6%	Survey and interview (n=81)
Kitinoja et al. [19]	Nigeria	2019	Fruits and vegetables	Post harvest (all) Wholesale Retail	14.9% 23.3% $\left[10\%,30\%\right]$	Commodity Systems Assessment Methodology (CSAM): literature review, interviews written questions, observations and direct measurement (10≤n≤15)
Kikulwe et al. [20]	Ouganda	2018	banana	Farm Collector Wholesale Retail	5% 7% 14% 18%	Interviews (farmers n=100, wholesalers n=10, collectors n=17, retailers n=40)
Kitinoja and AlHassan [12]	Ghana Rwanda	2012 2012	Cabbage Banana	Wholesale Retail	35.1 ± 33.1% 30.1 ± 24.4%	Direct measurements (cabbage n=30 samples (10 units per sample) Banana n=30 samples (20 units per sample))

## Data Availability

The bibliographic details of the 334 included studies are listed in the searchable database is available at https://PHCeres2030.net/ (accessed on 11 December 2021) and on the bibliography of this article. The code and additional data are available upon request.
